# A novel flow-cytometric based method to assess post-HSCT donor chimerism exploiting RNA hybridization

**DOI:** 10.1038/s41409-023-02143-9

**Published:** 2023-11-07

**Authors:** Silvia Nucera, Marco M. Sindoni, Cristina Bugarin, Tiziana Villa, Andrea Biondi, Adriana Balduzzi, Giuseppe Gaipa

**Affiliations:** 1 Tettamanti Center and Pediatrics, Fondazione IRCCS San Gerardo dei Tintori, Monza, Italy; 2 School of Medicine and Surgery, University of Milano Bicocca, Milan, Italy

**Keywords:** Translational research, Haematopoietic stem cells

## Abstract

Analysis of donor-recipient chimerism after hematopoietic stem cell transplantation (HSCT) is of pivotal importance for patient’s clinical management, especially in the context of mixed chimerism. Patients are routinely monitored for chimerism in sorted subsets of peripheral blood cells. However, measurement of chimerism in sorted immune cell subsets is technically challenging and time consuming. We here propose a novel, flow cytometry-based approach to detect donor cell chimerism in sex-mismatched HSCT. We exploit RNA PrimeFlow™ system, based on RNA hybridization, to detect mRNA from a lysine demethylase encoded by Y chromosome, KDM5D. This approach allows to distinguish male and female derived cells with around 1% sensitivity. The procedure can be coupled with multiparametric immunophenotyping to assess chimerism in specific immune cell subsets without the need for prior FACS-sorting. We apply this method to a cohort of HSCT patients (*n* = 10) and we show that it is consistent with standard PCR-based method. We also show that different T lymphocyte subsets display variable degrees of donor chimerism, especially in CD8+ T cell compartment where we observe an enrichment for recipient chimerism in central memory T cells. This method can be exploited to advance current knowledge on immune reconstitution focusing on specific subsets avoiding prior FACS-sorting.

## Introduction

Chimerism analysis is of pivotal importance after hematopoietic stem cell transplantation (HSCT) as it allows to evaluate engraftment of donor cells. From a technical standpoint, the gold standard is to assess chimerism by PCR technique, in particular short tandem repeat PCR (STR-PCR), a technique which however allows only limited sensitivity (1–5%) [1, 2]. In non-malignant diseases, such as sickle cell disease (SCD) or thalassemia, mixed chimerism is often observed since the development of reduced intensity conditioning regimens [3, 4]. In this context, a degree of disease-specific donor chimerism can still be sufficient to relieve the clinical phenotype but deserves close follow-up and specific therapeutic interventions. To this aim, it has been established that lineage-specific chimerism assessment is more predictive than total chimerism both in the context of malignant and non-malignant diseases and is therefore recommended for clinical management of patients [5–8]. However, chimerism on specific fractions can only be performed after FACS-sorting or magnetic beads sorting of subsets of interest. This is technically challenging, expensive and time consuming and, in case of FACS-sorting, it requires adequate instruments and trained personnel.

To overcome these limitations, we develop a flow cytometry-based system that allows to detect donor cell chimerism without the need for prior sorting. This method is also compatible with multicolor surface immunophenotyping, thus allowing to dissect donor cell chimerism in very specific immune cell subsets.

## Materials and methods

### Patients

Primary peripheral blood samples were obtained from pediatric patients who underwent HSCT and follow-up at Pediatric Hematology Department of IRCCS San Gerardo dei Tintori, Monza, Italy. All parents/guardians provided written informed consent. All analyses presented were performed on leftovers from routine clinical assessments and no extra timepoints were required. Patients’ characteristics are presented in Table 1. Of the 10 analyzed patients, 6 were transplanted for SCD, 2 for Beta-thalassemia (Beta-thal), 1 for Mucopolysaccharidosis I (MPS1) and 1 for acute lymphoblastic leukemia (ALL). Concerning donor type, 7 patients were transplanted from matched sibling donor, 1 from mismatched donor and 2 from matched unrelated donor. We analyzed timepoints from 2 months after HSCT to 58 months according to the patients.

**Table 1:** Patients’ characteristics, including indication for HSCT, donor, conditioning regimen and GvHD prophylaxis are summarized in the table.

Patient code	Indication HSCT	Recipient sex	Donor sex	Donor type	HSC Source	Conditioning regimen	GvHD prophylaxis	Rituximab	Months post-HSCT
1	SCD	F	M	MSD	BM	Treo + Flu + TT	Csa, MTX, ATLG	yes	3,4,5
2	Beta-thal.	M	F	MSD	BM	Treo + Flu + TT	Csa, MTX, ATLG	no	2,3,4
3	SCD	F	M	MSD	BM	Treo + Flu + TT	Csa, MTX, ATLG	yes	4,5,6,7,10
4	SCD	F	M	MSD	BM	Treo + Flu + TT	Csa, MTX, ATLG	yes	15.16
5	SCD	F	M	MSD	BM	Treo + Flu + TT	Csa, MTX, ATLG	yes	7,8,12
6	ALL	M	F	MMD	BM	TBI + VP16	Csa, MMF, PTCy	no	2.4
7	SCD	F	M	MSD	BM	Treo + Flu + TT	Csa, MTX, ATLG	no	38
8	MPS-1	M	F	MUD 10/10	BM	Treo + Flu + TT	Csa, mPDN, ATLG	yes	8
9	Beta-thal.	M	F	MUD 10/10	BM	Treo + Flu + TT	Csa, MTX, ATLG	yes	58
10	SCD	F	M	MSD	BM	Treo + Flu + TT	Csa, MTX, ATLG	no	1

Indication for HSCT: *SCD* sickle cell disease, *Beta-thal* beta thalassemia, *MPS-1* Mucopolysaccharidosis I, *ALL* acute lymphoblastic leukemia. Donor type: *MSD* matched sibling donor, *MUS* matched unrelated donor, *MMD* mismatched donor. *BM* bone marrow. Conditioning regimens: Treo+Flu+TT: treosulfan + fludarabine + thiotepa; TBI + VP16: total body irradiation + etoposide. GvHD prophylaxis: *Csa* cyclosporin, *MTX* methotrexate, *ATLG* anti-T lymphocyte globulin, *MMF* mycophenolate, *mPDN* methylprednisolone, *PTCy* post-transplant cyclophosphamide.

### Primeflow RNA assay and flow cytometry

To detect RNA in cells via flow cytometry we exploited Primeflow RNA assay (Thermofisher, Waltham, Massachusetts, USA. Catalog number 88-18005-210). KDM5D probe design was optimized to increase resolution and signal intensity. Briefly, after a first attempt with a single probe, we performed a 1:1 mixture of two different probes targeting KDM5D mRNA, both conjugated with Alexa647 (assay ID VA1-16845 and VPKA3GJ). RPL13A (assay ID VA4-13187) conjugated with Alexa 488 was used as internal control to assess the efficiency of hybridization reaction as suggested by manufacturer. Surface staining was performed prior to fixation and permeabilization according to manufacturer’s instructions. Antibodies used for surface staining are listed in Supplementary Table 1. Staining for B cell subsets was performed modifying the DURAClone IM B Cells tube (Beckman Coulter, Indianapolis, Indiana, USA) panel.

Primeflow RNA procedures were performed in accordance with the manufacturer’s instructions. Briefly, on the first day, after surface staining with directly conjugated antibodies, cells are fixed and permeabilized. After these steps, hybridization of the probes to target mRNA occurs at 40 °C for two hours. Samples are stored overnight at 4 °C in wash buffer with RNAse inhibitor (provided in the kit). Subsequently, on the second day, a pre-amplification step and amplification step are performed at 40 °C to create a branched-DNA structure as shown in Supplementary Fig. 1A. The final step is signal amplification to allow detection by flow cytometry.

Flow cytometric analysis was performed using 5-laser Aurora (Cytek, Fremont, California, USA). Data analysis was performed exploiting Spectroflo software (Cytek, Fremont, California, USA). For the analysis of KDM5D+ cells, besides surface staining, we only considered cells that were positive for RPL13A as internal control. This allowed us to focus only on cells where the hybridization occurred (Supplementary Fig. 1A).

FACS sorting of healthy controls and CD8+ subsets was performed using BD FACSAria I (Fondazione M. Tettamanti, Monza, Italy). FACS sorting of KDM5D positive cells was performed using BD FACSAria Fusion (Ospedale San Raffaele, Milan, Italy).

Purification of CD8+ and CD8+ naïve T cells was performed with CD8 microbeads and naïve CD8 T cell isolation kit (Miltenyi, Bergisch Gladbach, Germany) according to manufacturer’s instructions.

### DNA extraction

DNA from patients’ sample for STR-PCR analysis was extracted using Wizard Genomic DNA purification kit (Promega, Madison, Wisconsin, USA) according to manufacturer’s instructions.

DNA from sorted KDM5D+ cells was extracted using QuickExtract FFPE DNA extraction kit according to manufacturer’s instructions. DNA from sorted healthy donor cells or CD8 + T cells was extracted with QIAMP DNA Mini kit (Qiagen, Hilden, Germany).

### STR-PCR

STR-PCR was performed for diagnostic purposes according to international guidelines [1, 2, 9, 10]. PowerPlex 16 HS system (Promega, Madison, Wisconsin, USA) was used. PCR was performed according to manufacturer’s instructions.

### Digital droplet PCR

To perform digital droplet PCR (ddPCR) to evaluate KDM5D expression RNA was extracted with miRNeasy micro kit (Qiagen, Hilden, Germany) according to manufacturer’s instructions. RNA quantification was performed using Qubit (Invitrogen, Waltham, Massachusetts, USA) RNA BR Assay. 10 ng of RNA were used for PCR with One-Step RT ddPCR advanced kit for probes (Biorad, Hercules, California, USA). Primers for KDM5D FAM (dHsaCPE5032220) and HPRT1 HEX (dHsaCPE5192872) were purchased from Biorad.

### Statistical analysis

Data were summarized as mean ± SEM, or mean ± SD depending on data distribution. Statistical analyses were conducted using GraphPad Prism 9 software (GraphPad Prism, California, USA) according to data characteristics and are indicated in each figure. *P* < 0.05 was considered to be statistically significant.

## Results

### Technical validation of the flow cytometric chimerism probe

**Fig. 1:**
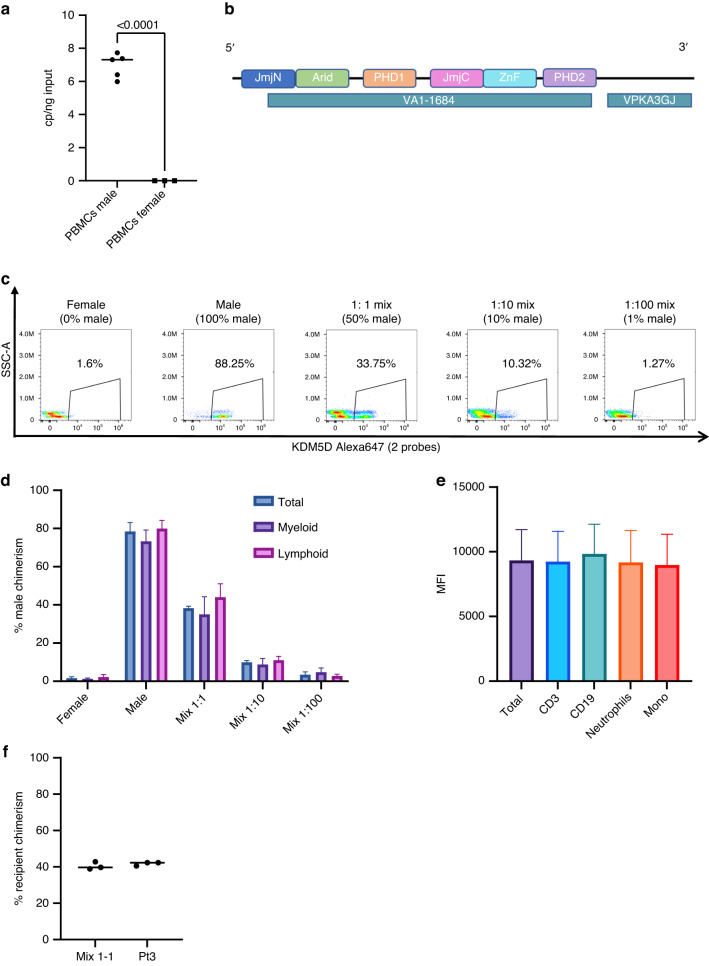
Establishment of a flow-cytometry based method to assess chimerism exploiting RNA hybridization on KDM5D. **a** Expression of KDM5D assessed by ddPCR in peripheral blood mononuclear cells (PBMCs) from male and female healthy donors (*n* = 5 and 3 respectively) confirms the presence of KDM5D mRNA only in male samples. **b** Scheme with mRNA KDM5D structure showing the regions where the two probes hybridize. **c** Representative FACS plot showing validation of KDM5D dual probe system on healthy donors. Mixing of samples was performed on white blood cell (WBC) counts with serial dilution of male sample with female cells. *n* = 4 independent experiments from a total of eight healthy donors. **d** Percentage of male chimerism from female, male and male:female mixed samples expressed as percentage on total CD45 + RPL13A+ live cells. **e** Mean fluorescence intensity (MFI) of KDM5D Alexa647+ cells on total CD45+ cells and in main immune cell subsets from male healthy donor (*n* = 4). **f** Graph showing technical reproducibility of three independent 1:1 mixes of the same male and female samples and of the same peripheral blood sample of a transplanted patient with mixed chimerism. Coefficient of variation was calculated for the 1:1 mix (0.051) and for the analyzed patient (0.024).

We aimed at developing a flow cytometry-based method to assess donor chimerism, so we decided to employ a commercially available system based on RNA hybridization that allows detection of mRNA (Primeflow™ RNA Assay, Thermofisher™) (Supplementary Fig. 1A). To develop a platform that could be used on a wide cohort of patients, we decided to exploit sex mismatch, which allows a broad application to HSCT patients. We first screened a list of potential candidate genes encoded by Y chromosome and scored them according to the degree of sequence similarity between the gene encoded by Y chromosome and its X-chromosome counterpart and according to the levels of expression in hematopoietic tissues in publicly available databases (Supplementary Fig. 1B) [11, 12]. The best candidate gene according to these parameters was KDM5D, a Y-chromosome encoded lysine demethylase containing zinc finger domains, which showed <90% sequence similarity (to maximize probe specificity) and intermediate expression levels in bone marrow (Supplementary Fig. 1B). We then assessed by ddPCR the expression of our target gene in immune cell subsets from male and female healthy donors and confirmed a good level of expression of KDM5D in male cells from several subsets such as total peripheral blood mononuclear cells (PBMCs), sorted CD3+ T lymphocytes, CD19+ B lymphocytes and in myeloid cells (Fig. 1a and Supplementary Fig. 1C). As aforementioned, we exploited an already available commercial system for RNA hybridization [13] but we obtained only limited sensitivity with standard commercial probe (Supplementary Fig. 1D). Therefore, we developed a dual-probe system to detect the expression of KDM5D by flow cytometry (Fig. 1b). With this dual probe system, we were able to efficiently detect the presence of male cells in serial dilution mix with female cells with around 1% sensitivity (Fig. 1c). We observed a good technical reproducibility with a coefficient of variation of 2–5% on total chimerism tested in three technical replicates from a mix of male and female cells and from a transplanted patient with mixed chimerism (Supplementary Fig. 1E). Furthermore, we were able to assess the presence of male cells in the main immune cell subsets (Fig. 1f). We also compared mean fluorescence intensity (MFI) in the different subsets of male cells and we found no significant differences in the expression of KDM5D, confirming the results obtained by ddPCR (Fig. 1e). Finally, we calculated stain index [14] for positive male and negative female samples (*n* = 3) and it resulted 2.9.

### Validation with clinical samples, comparison with PCR and dissection of chimerism among immunological subsets

**Fig. 2:**
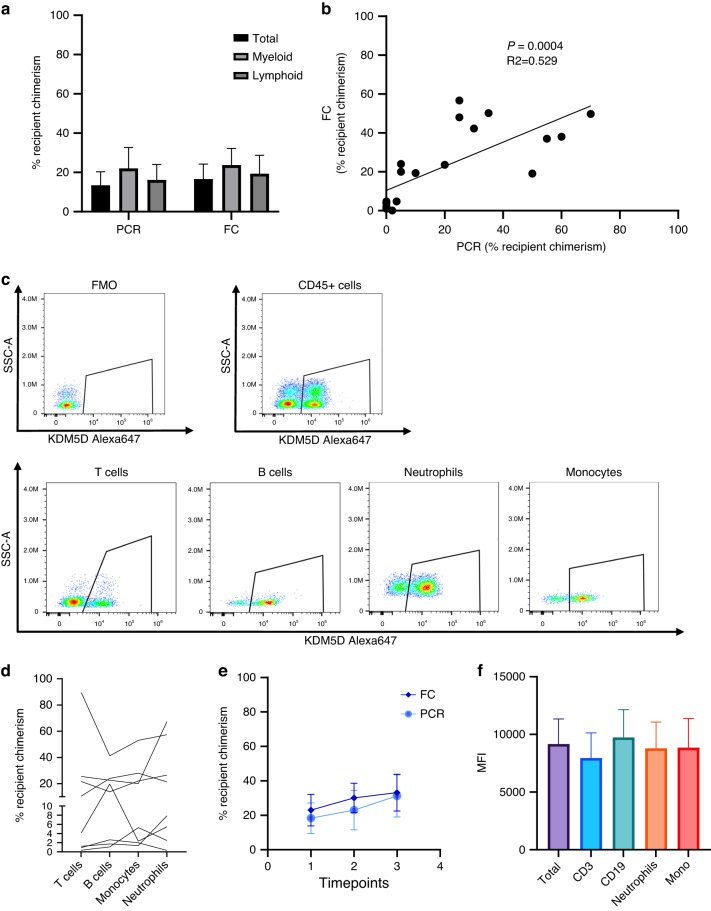
Validation of flow-cytometry based chimerism on HSCT patients. **a** Comparison of total chimerism in HSCT patients (*n* = 8) performed by STR-PCR (PCR) and RNA Prime flow (FC). Mean ± SEM. **b** Correlation between the levels of total recipient chimerism detected by PCR and flow cytometry (*n* = 19 samples. R^2^ = 0.529, *p* = 0.0004). **c** Representative FACS plot showing the presence of two distinct populations of cells from donor (in this case male) and recipient (female) origin in the different immune cell subsets and negative control (FMO). **d** Percentage of recipient chimerism in HSCT patients (*n* = 8) in the different immune cell subsets. **e** Kinetics of recipient chimerism detected by STR-PCR and flow cytometry at different timepoints on total peripheral blood samples shows a similar trend. **f** MFI of KDM5D Alexa647+ cells on total CD45+ cells and in main immune cell subsets in analyzed patients (*n* = 7) shows similar levels of expression in the different subsets. Mean ± SEM.

After this technical validation, we decided to test the system on a cohort of 10 pediatric HSCT patients with various degree of mixed chimerism along the follow-up. Patients’ characteristics are presented in Table 1. Briefly, 9 patients were transplanted for non-malignant diseases and 1 was transplanted for ALL. Recipient cell chimerism assessed via STR-PCR in our cohort ranged from 0 to 70% (median 15%). As shown in Fig. 2a, b, we obtained a significant correlation between standard STR-PCR and flow cytometry-based chimerism (recipient chimerism range 0–56%, median 20%). By combining RNA hybridization with surface staining, it was possible to distinguish donor vs recipient cells and to quantify the latter within the main immune cell subsets (Fig. 2c, d). Furthermore, we observed good degree of reproducibility over time (i.e., intra-patient variability) and, of note, a similar longitudinal trend between PCR and flow cytometry measurements (Fig. 2e, Suppl Fig. 2A). As for normal samples, we demonstrated, also in HSCT-derived samples, that MFI of KDM5D does not change significantly among the studied immune cell subsets (Fig. 2f, Supplementary Fig. 2B–D).

**Fig. 3:**
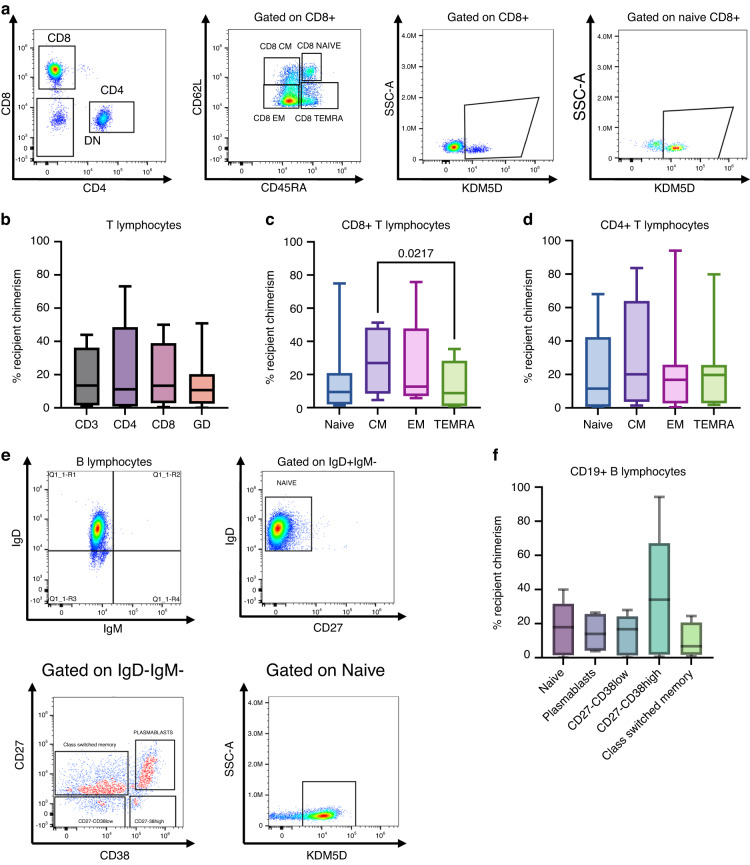
Application of flow-cytometry based chimerism to the study of immune reconstitution. **a** Representative FACS plot and gating strategy for T cell analyses (plots on the left) and representative expression of KDM5D on total CD8+ and naïve CD8+ T cells (plots on the right). **b** Recipient chimerism in CD3+ T cell subsets (*n* = 7) shows no significant differences among the main subsets. Repeated measures ANOVA with multiple comparison, mean ± SEM. **c** Recipient chimerism in CD8+ T cell subsets (*n* = 7) shows a significantly higher recipient chimerism in central memory T cells (CM) compared to terminally differentiated CD45RA+ cells (TEMRA). Repeated measures ANOVA with multiple comparison, mean ± SEM. **d** Recipient chimerism in CD4+ T cell subsets (*n* = 7). Repeated measures ANOVA with multiple comparison, mean ± SEM. **e** Representative FACS plot and gating strategy for B cell analysis and expression of KDM5D on naïve B cells. **f** Percentage of recipient chimerism in B cell subsets (*n* = 5). Only subsets with adequate number of events were taken into account for chimerism analysis. Mean ± SEM.

To further exploit the potential of this platform, we decided to expand our analyses to lymphocyte subsets thus correlating the function of lymphocytes with recipient chimerism (Fig. 3a). We observed no significant differences in the percentage of recipient cells among the main CD3+ T cell subsets such as CD4+ , CD8+ and Gamma Delta T lymphocytes (Fig. 3b). We then quantified the degree of recipient chimerism within CD4+ and CD8+ T cell memory profile (Fig. 3c, d and Supplementary Fig. 3) and we observed that central memory T cells (CM) tend to have a higher degree of recipient chimerism as compared to terminally differentiated cells (TEMRA); particularly in CD8+ T cells. Furthermore, we found that in both CD4+ and CD8+ T cells, the MFI expression level of KDM5D was similar among the subsets (Supplementary Fig. 3C, D).

In parallel, we also performed an analysis of B cell subsets and we did not observe significant differences among the subsets (Fig. 3e, f). We wondered whether pre-HSCT Rituximab might impact on B cell chimerism but we did not observe a significant difference in B cell chimerism (Supplementary Fig. 3I). Of note, we could verify that none of the patients in the cohort was treated with Rituximab after HSCT.

### Validation of KDM5D probe in sorted cells

To confirm that the different percentages of KDM5D+ cells corresponded to different degrees of recipient chimerism, we sorted CD8+ T cell subsets from patient #1 (female patient with male donor) and we performed ddPCR to assess the expression of KDM5D and STR-PCR on sorted cells. As shown in Supplementary Fig. 3E–G, we observe an inverse correlation between chimerism and KDM5D expression and comparable results between flow-cytometry and STR-PCR as in the other analyzed populations.

We also analyzed the expression of KDM5D in CD8+ naïve T cells from male and female healthy donors, and from HSCT patients (Supplementary Fig. 3H) confirming that HSCT patients express KDM5D proportionally to their mixed chimerism.

In conclusion, our data show that analysis of HSCT chimerism by RNA PrimeFlow™ system is feasible, reproducible, and correlate with standard STR-PCR method. Furthermore, by combining this system with flow cytometry immunophenotyping, it is possible to dissect the kinetics of chimerism within specific immune cell subsets without the need of prior FACS-sorting. Besides, from a biological perspective, this study highlights for the first time the potential impact of dissecting the specific contribution of different T lymphocyte subsets in the context of mixed chimerism during post-HSCT reconstitution.

## Discussion

In the present study we showed that it is feasible to exploit RNA hybridization to reproducibly assess donor cell chimerism by flow cytometry in HSCT patients. As a proof of principle, we exploited sex mismatch, as routinely done also for standard STR-PCR, which allows direct application of the method to a high percentage of patients independently from the degree of mismatch between donor and recipient, differently from previously reported flow cytometric methods [15]. So far, chimerism analysis on specific subsets has been shown to have important prognostic implications [3, 4]. Indeed, the main advantage of this platform is that it allows direct analysis of chimerism at single-cell resolution in specific hematopoietic subsets avoiding prior FACS sorting dramatically, thus abating the costs of such analysis (around 10-fold less). Furthermore, from a biological standpoint, this platform could be exploited to finely dissect the kinetics of lymphocyte reconstitution, particularly in the context of mixed chimerism, paving the way to biological studies on the kinetics of donor cell reconstitution and the impact of the diverse treatments (e.g., donor lymphocyte infusion) on the process.

The main limitation of the here-presented method is the sensitivity which is around 1%, similarly to STR-PCR. However, combination with specific surface markers (e.g., specific immunophenotype) could improve its sensitivity.

Moreover, although analysis on sex-mismatch does not apply to all HSCT patients, in case of same sex donor-recipient HSCT it would be possible to extend this platform by designing specific probes for HLA genes if a sufficient degree of mismatch is present. However, though not applicable to all HSCT patients, our platform could still be useful for several clinical and biological applications. Previous attempts to exploit flow cytometry for chimerism analysis were performed with anti-HLA antibodies by Schumm and collaborators [15]. Similarly to our method, those authors showed that flow cytometry allows multiple approaches (from analysis of cellular subsets to minimal residual disease). However, commercially available anti-HLA antibodies would apply to a minority of patients and customizing antibody for each donor-recipient pair would troublesome and expensive.

In our cohort, despite the small number of patients, we observed a good quantitative correlation between STR-PCR and flow cytometry, although with some limitations. However, kinetics of longitudinal monitoring over time was reproducible between the two methods. Indeed from a clinical standpoint [16] longitudinal kinetics is far more relevant than a single value, to decide post-HSCT clinical interventions.

Besides analyzing the recipient cell chimerism on myeloid and lymphoid subsets, as currently done in clinical routine, we were able to combine chimerism analysis with more specific immunophenotypic antibody panels, in particular for T and B lymphocytes. The finding that in our cohort of patients, despite limited, central memory T cells and in particular CD8+ central memory T cells, showed a higher degree of recipient chimerism, is compatible with data from mouse models and solid transplant recipients after anti-T lymphocyte globulin (ATLG) treatment [17, 18]. We could not assess this aspect in our cohort as 9 patients analyzed received ATLG as GvHD prophylaxis and the only patient who did not receive ATLG (patient #6) developed a full donor chimerism after the first two months post-HSCT. However, such observation should be considered a novel finding for HSCT patients and paves the way for ad hoc studies to assess the impact of recipient chimerism in central memory T cells.

In conclusion, we here provide a proof of principle that RNA expression can be efficiently exploited to detect chimerism by flow cytometry in sex-mismatched HSCT and that this approach allows to abate the costs and expand the potential of chimerism analyses.

### Supplementary information

Supplemental Data
