# Supplementary material for: A novel flow-cytometric based method to assess post-HSCT donor chimerism exploiting RNA hybridization
Source: Bone Marrow Transplant. 2023 Nov 7;59(2):171–7. doi: 10.1038/s41409-023-02143-9 (PMC10849949; doi:10.1038/s41409-023-02143-9)
Supplement: Supplementary file 1 — Supplemental Data [file 41409_2023_2143_MOESM1_ESM.docx]

**SUPPLEMENTARY TABLES**

**Supplementary Table 1**

Antibodies against surface markers used for the immunophenotypic analysis are listed in the Table below.

| **Antigen** | **Manufacturer** | **Code** | **Fluorochrome** |
| --- | --- | --- | --- |
| CD3 | BD | 558117 | PB |
| CD19 | Beckman | IM3628 | PC7 |
| CD14 | Biolegend | 301805 | PE |
| CD45 | BD | 655873 | V500 |
| CD16 | BD Horizon | 563690 | BV786 |
| IgM | Biolegend | 314522 | BV510 |
| IgD | BD | 562024 | PE |
| CD38 | Beckman | B49200 | APC ALEXA750 |
| CD27 | BD | 562513 | BV421 |
| CD24 | BD Horizon | 563720 | BV650 |
| CD8 | BD Horizon | 562282 | PECF594 |
| CD62L | Biolegend | 304834 | BV605 |
| CD45RO | BD Horizon | 563722 | BV711 |
| TCR GD | BD | 655410 | PC7 |
| ZOMBIE | Biolegend | 423105 | NIR |
| CD4 | BD | 641398 | APCH7 |

**SUPPLEMENTARY FIGURES**

**Supplementary Figure 1**

(A) The scheme shows how the procedure allows to detect by flow cytometry mRNAs present in the cells. The figure was created using BioRender.com (B) Expression of a selection of genes encoded by Y chromosome according to Human Protein Atlas (https://www.proteinatlas.org/) (left) and (right) degree of similarity between male and female counterpart as reported by Godfre et al. (11). (C) Expression of KDM5D assessed by ddPCR in sorted CD3+, CD19+ and myeloid cells from male healthy donors (n=2) compared to total PBMCs (n=5). (D) Representative FACS plot showing the lack of difference of expression between male and female samples with a single probe for KDM5D. (E) Sorting strategy for KDM5D positive cells from patient #2 (left). Graph on the right shows results from STR-PCR comparing the levels of recipient chimerism in total peripheral blood and sorted KDM5D+ cells at the same timepoint. Cells positive for KDM5D are 100% derived from male recipient.

**Supplementary Figure 2**

(A) Comparison of percentage of recipient chimerism detected by STR-PCR and flow cytometry (FC) in each single patient (n=8). For patients #1,2,3 and 4 multiple timepoints are available with both methods and show similar kinetics. (B) MFI of KDM5D Alexa647+ cells on total CD45+ cells and in main immune cell subsets in each analyzed patient (n=7). Graph shows mean ± SEM (C) Table shows the mean, median, standard deviation (SD) and coefficient of variation (CV) among the main immune cell subsets in 8 patients. (D) Table shows the consistency of MFI among the different subsets within each analyzed patient. While inter-patient variability is higher, intra-patient variability is dramatically reduced.

**Supplementary Figure 3**

Chimerism analysis in CD4+ (A) and CD8+ (B) T cell compartment for each analyzed patient expressed as percentage. Analysis of Mean Flourescence Intensity (MFI) of CD4+ (C) and CD8+ (D) subsets shows no differences in the levels of expression of KDM5D. (E) KDM5D expression by ddPCR in sorted CD8+ subsets from patient #1 shows higher levels of expression in naïve T cells compared to the other subsets. Consistently, flow-cytometry based chimerism from CD8+ subsets from the same patient shows a lower degree of recipient chimerism in naïve T cells (F). (G) STR-PCR shows comparable levels of chimerism on the same subsets. (H) KDM5D expression in CD8+ naïve T cells from male healthy donors (n=3), female healthy donor (n=1) and HSCT patients (n=5) shows how HSCT patients express levels in between purely male and purely female samples in a proportionate way to % of male cells in the sample. (I) Ratio between B cell recipient chimerism and total recipient chimerism in patients treated or not with Rituximab shows no differences (n=5). Mean ± SEM.
